# Broiler Chickens On-Farm Welfare Assessment: Estimating the Robustness of the Transect Sampling Method

**DOI:** 10.3389/fvets.2019.00236

**Published:** 2019-08-06

**Authors:** Neila BenSassi, Xavier Averós, Inma Estevez

**Affiliations:** ^1^Department of Animal Production, Neiker-Tecnalia, Vitoria-Gasteiz, Spain; ^2^IKERBASQUE, Basque Foundation for Science, Bilbao, Spain

**Keywords:** broiler, chicken, welfare, assessment, transect, method, robustness

## Abstract

Assessing commercial broiler chickens' welfare usually comes at the cost of reduced precision due to the large flock sizes and required time commitments. The transect method for on-farm welfare assessment is conducted by walking within delimited paths between feeder and drinker lines within the commercial house, referred to as transects. This non-invasive method is conducted by detecting birds with signs of impaired welfare indicators, which include leg problems, sickness, body wounds, and feather dirtiness. The transect method has been validated for commercial turkey flocks but not for broiler chickens due to the large flock sizes. The aim of this study was to evaluate the robustness of the transect method in broiler chicken flocks through a capture–recapture approach of a known subpopulation of 80 birds. Groups of 10 chickens were captured and individually marked in eight locations of the house. Two observers collected the number and position of the detected marked birds while walking along non-adjacent transects (four samplings/house/day) during the two following days. Detection and repetition rates per house, and within transects, were calculated, as well as the effects of flock density, transect number/house (six vs. eight), and sampling time (morning vs. afternoon). The number of traveled transects was calculated for birds detected more than once, and the population random distribution was tested by comparing the number of observed and expected birds/transect. Results showed more than 64% of detection rate with a repetition rate/house sampling of 24% and per transect of 1.66%. Higher repetition rates in six-transect houses and during morning samplings were detected. The number of traveled transects was higher in eight-transect houses and from birds first detected at walls, indicating longer traveled distances in wider houses. In addition, bootstrapping techniques were used to calculate the optimal sampling effort. Our findings indicate that the lowest repetition rates and optimal sampling can be achieved by assessing two transects, being one wall and one central, separated by three transects in between. Such sampling procedure would provide robust results for welfare assessment of commercial broiler chicken flocks.

## Introduction

Public concern about animal welfare, among other reasons, has resulted in the need of developing assessment protocols that can be applied on commercial farms to provide consumers with information on certain welfare requirements. The Welfare Quality^®^ protocols (1) are the most commonly used welfare assessment methods for cattle, pigs, and poultry. Regarding poultry, the protocols are time-consuming despite the limited sample size that is assessed (2). Attempts to simplify the protocols included the evaluation of on-farm provided resources (3) or the assessment of postmortem condition (4). The use of technology to assess broiler welfare through precision livestock farming is also emerging (5), even though many of these methods are still at the experimental phase (6).

The transect method was recently developed for on-farm welfare assessment of meat poultry (7, 8). This method is implemented by walking within transects, which are defined as the areas delimited by feeder and drinker lines while collecting the prevalence of birds showing meat poultry welfare issues. Such issues include leg problems (lame and immobile birds), sickness (including sick and terminal birds), skin wounds (head, back, or tail wounds), and/or feather dirtiness [(7, 9, 10) for detailed definition of welfare indicators]. The transect method is non-invasive and efficient as the method does not require bird capture or manipulation. Data are collected by clicking on the i-WatchBroiler app assessment screen (11) each time a bird showing one of the listed indicators is detected within the assessed transect.

The transect method was validated for commercial turkeys by comparing the results with the individual assessment of the entire flock during load out (8). However, the much larger group size of commercial broiler chicken flocks impedes the same type of validation. Previous studies tested the sensitivity of the transect method to detect effects of bird and house features along with environmental enrichment (9, 10). On-farm welfare problems detected with the transect method correlated with increased rejections at slaughter (9, 10), highlighting the link between on-farm collected data and production outcomes. These results suggest the robustness of the transect method, even though there are still questions regarding its accuracy. In this sense, it is still unknown whether assessments can truly detect all birds with problems and the extent to which results may be altered by repetition of birds.

An alternative approach for estimating the robustness of the method may be to track a known bird subpopulation to determine the detection rate along with possibilities of repetition. In wild life ecology studies, estimation of the population abundance and movement patterns is conducted using different marking and tracking methods (12). The capture–recapture method, which consists in marking individuals in a population, releasing, and recapturing (or resighting) them later on (13), may be a useful approach to estimate the movement of birds when conducting transects and particularly to estimate birds that may be repeated during the assessment process. As birds' movement is also related to management aspects (14), studying management features might provide insights on their potential effects on assessment results. For instance, broiler chickens are more active during the morning as compared to afternoons (15), which might increase the likelihood of overlooking birds during morning assessments or, on the contrary, could result in a higher rate of repeated birds if they move transversally. Birds' use of space is affected by stocking density (16, 17). Thinning, a commercial practice consisting in depopulating part of the flock, results in decreased stocking density, which may modulate movement patterns of the remaining birds (18) and increase the probabilities of overlooking individuals as more space is available. Moreover, birds are reported to travel longer distances when in large experimental pens (19, 20). According to the house dimensions and the flock density, birds might be overlooked as they tend to escape from observers by running in front or taking the perpendicular direction to the observer's movement. This could also result in repeating birds when conducting more than one transect/house. Repetition in both longitudinal and transversal directions (i.e., both in the evaluated and neighboring transects) should then be investigated. A higher use of enclosure peripheral areas was reported at higher stocking densities (21–23), especially for impaired birds (9). This suggests an uneven distribution of birds, indicating that both central and peripheral areas should be sampled when implementing transects.

The aim of this study was to assess the robustness of the transect method for broiler welfare assessment by determining its capability to detect individuals of a known subpopulation. The probability of repeating birds within and across transects was also tested. For this purpose, a subpopulation of broiler chickens was marked and then tracked for two consecutive days to estimate the detection and the repetition rates within and across transects (per house). We predicted higher detection and lower repetition rates with high densities and during lower activity periods. We also estimated the number of transects that repeated birds traveled, the subpopulation distribution, and the effects of tested management factors, expecting a higher number of traveled transects at lower flock densities and in larger houses.

## Materials and Methods

### Experimental Design and Data Collection

This study was conducted in Northern Spain from March 2016 to November 2017. Eleven commercial broiler flocks placed in three different farms were used in the study. All farms belonged to the same integrating company and followed identical management practices. House dimensions ranged from 1,250 to 1,950 m^2^ (Table 1) with initial stocking densities ranging from 17 to 19 birds/m^2^. All houses were provided with automatic drinkers, feeders, ventilation, and artificial light. Flocks were all of mixed genders. Genetic lines were Ross 308, Cobb 500, or a mix of both (Ross 308/Cobb 500). Thinning took place during the fifth week of age in some flocks. When assessments were performed, flock densities ranged between 11.36 and 17.84 bird/m^2^ (Table 1).

**Table 1:** Number of sampled houses per farm, house dimensions, number of transects per house, and stocking densities of sampled flocks at the time of the data collection.

**Farm**	**Houses/farm**	**House dimension (m^**2**^)**	**Transects/house**	**Rounds**	**House**	**Flock density^**a**^ (birds/m^**2**^)**
1	1	1,950	6	1	1	16.35
2	2	1,250	6	1	1	16.44
					2	16.40
				2	1	11.85
					2	11.90
				3	1	17.19
					2	17.83
3	2	1,500	8	1	1	17.52
					2	17.83
				2	1	12.82
					2	11.39

a *Stocking densities in the day of bird marking; lowest values correspond to thinned flocks prior to the start of the study*.

Before data collection, house dimension and transect width were measured using a laser meter (Robert Bosch GmbH, GLM 250 VF Professional, Switzerland). The length and width of the house were measured by placing the laser meter in one wall and measuring the distance to the opposite wall. Each area (or path) delimited by feeder and drinker lines was considered a transect if wider than 1 m. Transects were categorized as “wall transect” if delimited by a wall on one side and as “central transect” if delimited by feeder and/or drinker lines on both sides. Transect measurements were taken with the laser meter by two observers, with one observer maintaining the laser meter on a feeder/drinker line, while the second placed a clapboard on the next feeder/drinker line. The number of transects per house were either six or eight (Table 1) depending on the house width (10–15 m) and the disposition and number of feeder and drinker lines. In total, 4 six-transect and 3 eight-transect houses were used. Mean transect width was 1.83 ± 0.029 m [mean ± standard error (SE)], and the estimated mean number of sampled birds/transect was 3,150 ± 56 (mean ± SE).

**Figure 1:**
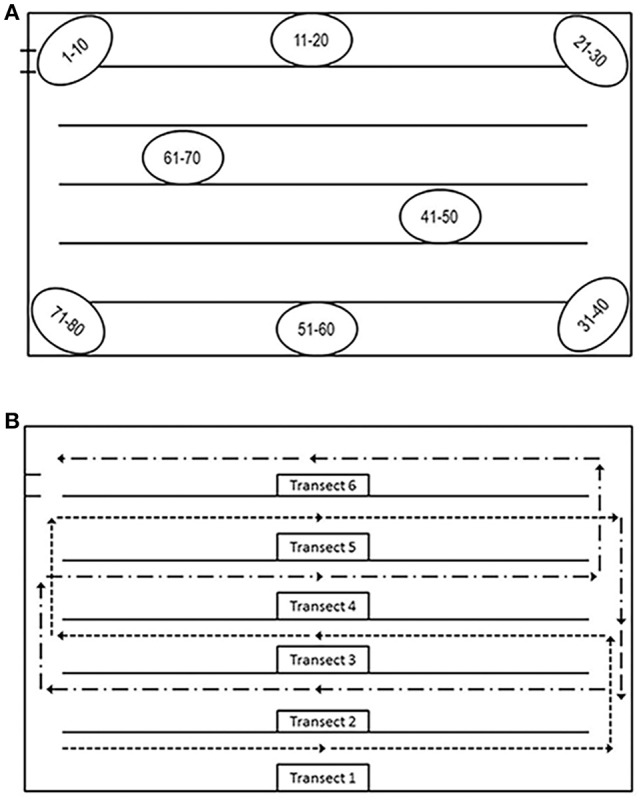
**(A)** chicken subpopulation marking distribution and **(B)** tracking pattern. The dashed lines starting in transects 1, 3, and 5 show the first part of the data collection, while those traveling transects 2, 4, and 6 show the second part.

The transect method is conducted by walking within two transects, in a similar way as a farmer would routinely check for the birds' health. Along such standardized walks, each observed bird showing any of the impaired welfare indicators (leg problems, sickness, wounds, and dirtiness) is recorded by clicking on the assessment screen of the i-Watchbroiler app. By knowing the width and length of each transect and the total bird population, the prevalence of each welfare indicator per transect is provided by the mobile app. In this study, however, our targeted bird subpopulation was composed of individually marked chickens collected and marked with numbers in different locations of the house. A total of 80 birds were captured at random at eight house locations. To maximize the distance and minimize disturbances for the birds, 10 chickens were marked per location (Figure 1A). Birds captured in a given house location were marked and released in the same area. All chickens were marked on the back of their head with numbers (1–80) for individual identification using a black permanent, nontoxic marker. Marking was performed at 30 ± 2 (mean ± SD) days of age. Twenty-four hours after marking, birds were tracked for two consecutive days by two trained observers who collected data in separated houses within the same farm. Although the evaluation of two transects is usually performed during commercial welfare assessments (7), for the purposes of this study, all transects of the house were assessed. Any of the 80 individually tagged birds found along each transect in the house was recorded in the corresponding transect, even those with repeated observations.

Observations were always performed following the recommendations of Marchewka et al. (7, 8) and starting from transect 1, located at the right side of the house, with the house entrance door as a reference (Figure 1B). As standard practice with the transect method, sampling of two adjacent transects was avoided in order to minimize repetition risks. Transect walks were conducted until completing all transects in the house by assessing transects 1, 3, and 5, and returning to assess transects 2, 4, and 6 (Figure 1B). During the transect walks, the identity and spatial location of detected marked birds were recorded on a house template that included longitudinal references and the location of all transects. Each of the two observers conducted the assessment simultaneously in one of the houses, swapping houses when finishing. A total of four samplings were collected, two per observer, house, and day, being two in the morning and two in the afternoon. A 15-min interval was allowed between house samplings. Data of one of the sampled houses were missing for the second day of data collection due to the thinning of the flock.

### Calculation of Parameters and Statistical Analyses

#### Detection and Repetition Rates

The detection rate per house sampling was calculated as $\left(\frac{\text{N detected marked birds per house sampling}}{\text{Total N of marked birds}}*100\right)$. The repetition rate/house sampling was calculated as $\left(\frac{\text{N repeated marked birds per house sampling}}{\text{N detected marked birds in the same house sampling}}*100\right)$. The bird repetitions within a transect were calculated as $\left(\frac{\text{N repeated marked birds in a transect}}{\text{N detected marked birds witin the same transect}}*100\right)$.

For repeated birds, the transversal movement was estimated by calculating the number of traveled transects between the first and second observations of each repeated bird in a house sampling. The percentage of repeated birds was calculated according to the distance, in transects, from the first observation. For instance, in a six-transect house, we calculated the percentage of repetition two transects away from the first conducted by summing the number of birds that were first detected in transect 1 and repeated in 3, first detected in 2 and repeated in 4, first detected in 3 and repeated in 5, and first detected in 4 and repeated in 6. This number was then divided by the total number of birds detected.

The effects of flock density, number of transects/house (six vs. eight transects), and sampling time (morning vs. afternoon) on detection and repetition rates/house sampling were tested assuming a Gaussian distribution. The same effects were tested for the repetition rate within transects, variable that was modeled assuming a Poisson distribution. Repeated-measures, generalized linear mixed model analyses of variance (ANOVAs) were carried out with the GLIMMIX procedure in SAS 9.3 (24). All effects were introduced as categorical variables except flock density, which was included as a covariate. The effect of the observation day was first included in the model and then removed due to non-significance. Flock nested within farm was included as a random effect, and the day-by-house sampling was the repeated-measures unit in the three models. A first-order autoregressive covariance structure was assumed to account for any linear dependence of flock measures over time.

For the number of traveled transects in the case of repeated birds, the effects of flock density, number of transects/house, and transect position where the bird was first detected (wall vs. central) were tested assuming a Gamma distribution. The sampling time (morning vs. afternoon) was first introduced in the model but then removed due to non-significance. The repeated-measures unit consisted in the interaction between the observer and observation day. For statistically significant effects (*P* < 0.05), least squares means differences were computed for all models, with *P*-values adjusted for multiple comparisons using Tukey tests.

#### Distribution of the Marked Subpopulation

To test the differences in distribution between the expected and the observed number of marked birds, we calculated the distribution index according to the formula by Keeling et al. (25): $\frac{{\left(\text{N observed marked birds in transect–N expected marked birds in transect}\right)}^{\text{2}}}{\text{N expected marked birds in transect}}$ The expected number of marked birds/transect was estimated according to the specific transect dimensions. The distribution index tends to be zero when the observed and expected number of marked birds are similar, indicating a random bird distribution. The distribution index was first calculated by transect, then we calculated a mean distribution index per flock and per day for wall and central transects.

The effects of number of transects/house (six vs. eight transects), transect position (wall vs. central), and their interaction were tested on the subpopulation distribution index assuming a lognormal distribution. Flock density at the day of sampling was first included in the model and then removed due to non-significance.

#### Bootstrapping Simulations

Bootstrap analysis was applied to examine the method's stability when varying the number of sampled transects per house. This method, used to optimize sampling methods, generates a collection of simulated random sampling combinations from the original data set using the Monte Carlo method (26) to construct the bootstrap distribution (27, 28). Expected mean and SE of the data set were calculated by taking random samples of one transect or combinations of two to five transects in six-transect houses, and two to seven transects in eight-transects houses. Simulations were run 10,000 times using the PROC SURVEYSELECT in SAS 9.3 (24) software. Calculations were averaged per farm (given that houses belonging to the same farm were of the same size) and across all rounds of data collection.

### Ethical Statement

This study complied with the Spanish legislation regarding the use of animals for experimental and other scientific purposes (Real Decreto 53/2013).

## Results

### Detection and Repetition Rates, and Subpopulation Distribution

The detection rate of the marked subpopulation was 64.76% ± 0.87 (mean ± SE), with no effect of any of the tested factors (Table 2). The repetition rate when conducting all transects per house was 23.85% ± 0.77, but was as low as 1.66% ± 0.58 (mean ± SE) within transect. The repetition rate/house was higher in six-transect as compared to eight-transect houses (Table 2), while higher repetitions within transects were found in morning samplings (Table 2).

**Table 2:** Effects of stocking density, number of transects/house (six vs. eight), and sampling time (morning vs. afternoon) on the detection and repetition rates per house and within transects (mean ± SE) of a marked subpopulation of broilers assessed using the transect method.

		**Variables**	**Detection rate^**b**^**	**Repetition rate/house sampling^**c**^**	**Repetition rate within transects^**c**^**
Flock density (birds/m^2^)		Mean RC^a^	0.232	−0.339	0.149
		SE	0.339	0.265	0.137
		*F*_(1, 16)_	0.47	1.63	1.26
		*P*	0.502	0.220	0.278
Transect number/house	6 transects	Mean^2^ (%)	63.735	26.405	1.389
		SE	1.166	0.915	0.537
	8 transects	Mean^2^ (%)	66.129	20.531	2.006
		SE	1.335	1.048	1.146
		*F*_(1, 16)_	1.82	17.84	0.59
		*P*	0.196	<0.001	0.452
Sampling time	Morning	Mean^2^ (%)	65.177	24.054	3.126
		SE	1.262	0.991	1.112
	Afternoon	Mean^2^ (%)	64.687	22.881	0.263
		SE	1.233	0.968	0.263
		*F*_(1, 16)_	0.17	0.72	5.03
		*P*	0.687	0.406	0.038

a *Mean RC: Mean regression coefficients estimated for the effect of flock density on detection and repetition rates per house sampling and within transect*.

b *For repetition rate within transect, P-values and F correspond to the results of the statistical model run with Poisson distribution, whereas mean and SE are calculated from raw data*.

c Detection rate = (number of marked birds detected by house sampling/total number of marked)*100; repetition rate per house sampling = (number of repeated birds during the house sampling/number of detected in the same house sampling)*100; repetition rate within transects = (number of repetitions in one transect/number of detected in the same transect)*100.

When only considering the observations of repeated birds, 67 and 71% of the repetitions occurred in adjacent transects in six- and eight-transect houses, respectively (Table 3). In both house sizes, the percentage of repetitions decreased as the number of transects in between increased. When considering the transect where each bird was first detected and the location where it was observed later on, results showed that, on average, birds traveled more than two transects if first detected in transect 1 (wall), while they traveled one to two transects if first detected in any of the other transects (Table 3).

**Table 3:** Distribution of repeated birds (%) according to the number of transects away, and number of traveled transects (mean ± SE) according to where marked birds were first detected.

**Distribution of repeated birds (%)**	**Transect number/house**		**Adjacent transect**	**2 transects away**	**3 transects away**	**4 transects away**	**5 transects away**	**6 transects away**	**7 transects away**
	6		71.212	15.151	10.038	2.841	2.272	-	-
	8		67.372	12.689	9.365	4.230	3.021	2.719	0.302
**N traveled transects**		**First detected in**	**Transect 1**	**Transect 2**	**Transect 3**	**Transect 4**	**Transect 5**	**Transect 6**	**Transect 7**
	6	Mean	2.055	1.319	1.715	1.360	1	-	-
		SE	0.105	0.077	0.104	0.046	0	-	-
	8	Mean	2.402	1.948	2.178	1.864	1.760	1.187	1
		SE	0.205	0.176	0.247	0.177	0.176	0.070	0

**Figure 2:**
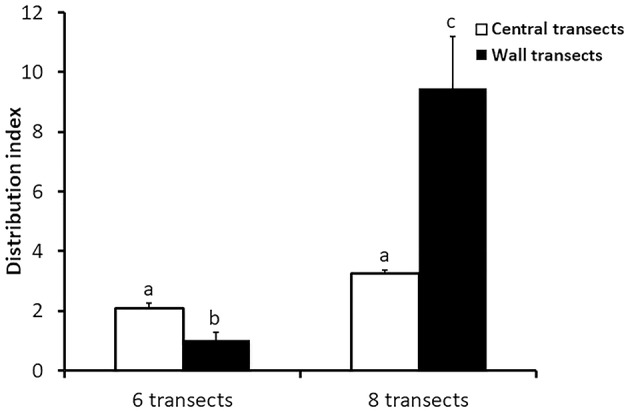
Interaction between transect number/house (six vs. eight) and transect position (central vs. wall) on the distribution index of a marked subpopulation of broiler chickens. [The distribution index was calculated as: (N observed marked birds in transect—N expected marked birds in transect)^2^/(N expected marked birds in transect)].

Regarding the subpopulation distribution, our results showed that the distribution index was lower in six-transect houses [*F*_(1, 37) =_ 14.43, *P* < 0.001]. Mean values are presented in Table 4. Significant differences were detected for the interaction between the number of transects/house and transect position [*F*_(1, 37) =_ 29.22, *P* < 0.001; Figure 2]. The number of traveled transects per repeated marked birds was higher in eight- compared to six-transect houses, and for birds initially detected on wall transects in comparison with those detected in central ones (Table 5).

**Table 4:** Mean ± SE of transect width, number of expected and observed marked birds per transect, and the distribution index in six and eight transect houses and according to the transect position (wall vs. central).

**Transect number/house**	**6 transects**				**8 transects**			
**Mean house width (m)**	**11**				**15**			
	**Mean**		**SE**		**Mean**		**SE**	
Transect width (m)	1.773		0.029		1.800		0.055	
Expected^*a*^	13.333		0.215		10		0.284	
Observed	10.802		0.236		7.945		0.251	
Distribution index	1.538		0.200		6.340		1.171	
**According to transect position**	**Central**		**Wall**		**Central**		**Wall**	
	**Mean**	**SE**	**Mean**	**SE**	**Mean**	**SE**	**Mean**	**SE**
Transect width (m)	1.850	0.039	1.618	0.034	2.171	0.062	1.136	0.034
Expected	13.957	0.305	12.086	0.133	11.350	0.319	5.950	0.182
Observed	9.972	0.267	12.821	0.381	6.594	0.231	12.00	0.432
Distribution index	2.060	0.216	1.018	0.274	3.252	0.104	9.429	1.771

a *Number of animals expected to be present in a transect according to the total number of birds present in the house during the evaluation and, the total width of the house, and the transect width*.

**Table 5:** Effects of stocking density, number of transects/house (six vs. eight transects), and transect position (wall vs. central) on the number of traveled transects over repeated observations of marked bird using the transect sampling method.

			**Traveled transects of repeated birds**
Flock density (birds/m^2^)		Mean RC^a^	−0.012
		SE	0.008
		F^b^	2.04
		P	0.153
Transect number/house	6 transects	Mean	1.580
		SE	0.046
	8 transects	Mean	1.881
		SE	0.068
		F^b^	16.21
		P	0.005
Transect position	Central	Mean	1.438
		SE	0.036
	Wall	Mean	2.068
		SE	0.087
		F^b^	56.91
		P	<0.001

a *Mean RC: mean regression coefficient estimated for the effect of stocking density on the number of traveled transects, and the marked population distribution index [CI = (number of observed – number of expected)^2^/number of expected]*.

b *The number of degrees of freedom was F_1, 848_ for the stocking density and F_1, 7_ for the number of transects/house and transect position*.

### Bootstrapping Simulations

The results of the bootstrapping simulations on the percentage of detected marked birds/m^2^ showed that the mean value remained stable irrespectively of the number of transects observed (Table 6).

**Table 6:** Bootstrapping simulation results for the percentage of marked birds detected/m^2^ (Mean ± SE) according to the number of transects assessed for each farm across sampled flocks.

		**Farm 1**		**Farm 2**		**Farm 3**			
Mean flock size (birds)		31,891		19,051		22,359			
House dimension (m^2^)		1,950		1,250		1,500			
**Covered area (%)**	**Transects number**	**Mean**	**SE**	**Mean**	**SE**	**% information**	**Transects number**	**Mean**	**SE**
17	1	0.037324	0.000111	0.051112	0.000185	12.50	1	0.042460	0.000217
33	2	0.037320	0.000079	0.050960	0.000126	25.00	2	0.042626	0.000154
50	3	0.037272	0.000063	0.051290	0.000104	37.50	3	0.042660	0.000125
66	4	0.037222	0.000055	0.051372	0.000092	50.00	4	0.042467	0.000107
84	5	0.037313	0.000049	0.051274	0.000082	62.50	5	0.042649	0.000096
100	6	0.03729	0.000045	0.051314	0.000075	75.00	6	0.042595	0.000089
						87.50	7	0.042739	0.000081
						100.00	8	0.042617	0.000076

## Discussion

The robustness of the transect method was tested by applying the capture–recapture approach on a known subpopulation of broiler chickens reared under commercial conditions. Eighty birds were individually marked and tracked across transects during two consecutive days. Detection and repetition rates per house and within transect were calculated. For repeated birds, we estimated the number of traveled transects. The hypothesized subpopulation random distribution was analyzed considering the transect number and position. The recommended number of transects to sample for a representative assessment with the minimum effort was estimated.

### Detection and Repetition Rates

The transect method intends to be a practical welfare assessment tool for meat poultry reared under commercial conditions. Detection rate of marked chickens, when all transects were observed, reached nearly 65% of the marked subpopulation. Given that detection rates only include detected non-repeated birds, individually locating almost two thirds of a subpopulation of 80 individuals within flocks that ranged between 15,000 and 32,000 birds can be considered quite satisfactory, especially if we consider the efficient and non-invasive features of the method. Because of the natural tendency of birds to move away when perceiving an approaching human, some of the marked birds might have been overlooked, even though walking through transects was always performed at a slow pace. Nevertheless, it is important to point out that the probability of detecting all 80 marked birds depends on the probability that the observer and each particular bird coincide in time and space in one of the assessed transects. This combination of likelihoods makes it statistically improbable to detect all specific marked birds in one round of house sampling, explaining why the detection rate when assessing the entire house is unlikely to reach 100%.

Almost 24% of the birds were repeated in the house samplings as all transects were conducted. Differences in bird movement patterns (29) may help in explaining the results obtained in the detection and repetition rates, as birds with higher mobility are likely to be repeated later on in one or more transects. Our results on the repetition rate/house sampling suggest that most birds tend to move away laterally as the observer walks along the transect. In fact, lateral movements at an angle of 90° from the potential predator's line of attack have been shown to be the natural escaping strategy in bird taxa (30). The tendency to move laterally to the observer trajectory explains the repetition rates obtained when assessing the entire house. Indeed, higher repetition percentages were found especially in transects adjacent to the one first conducted (see Table 3). On the contrary, it was particularly important to note that the repetition rate within transects (birds observed more than once in a particular transect) was low with a mean of 1.66 ± 0.58% (mean ± SE). This is important in practice, given that assessments are advised to be based on two transects per house (7), which means that chances of overestimating welfare problems are actually low.

The regression analyses showed that the risk of repetition within transects was higher in the morning assessments as compared to afternoon (Table 2). This difference is likely due to the higher morning activity levels reported for the domestic fowl (15), usually dedicated to forage (31). Even though the repetition rate within transects was overall low, higher activity levels in the morning may have resulted in some birds that did not move in a perpendicular direction but kept moving within the length of the transect or may have moved away and returned to the observed transect within a short time period. Higher repetition rates/house were also detected in six-transect in comparison to eight-transect houses. Because six-transect houses are narrower than eight-transect houses (11 vs. 15 m, respectively), the probability of observing the same bird increases as a result of the lower number of transects. Besides, when considering the sequence of observations in wider houses, the observer assessed transects 1, 3, 5, and 7 before coming back to transects 2, 4, 6, and 8 providing birds with a longer time lapse and space to redistribute, thus decreasing the risk of repetitions. In addition, our results on the percentage of repeated birds among transects showed very high repetition in adjacent transects in comparison to the following ones. These results not only support the recommendation by Marchewka et al. (7) of avoiding observations in adjacent transects but also confirm our suggestion to sample transects that are further away. According to our findings in Table 3, skipping at least three transects would minimize the risk of repetition. This is particularly advisable in narrower houses.

It is clear from our results that birds in wider houses (i.e., eight-transect houses) traveled more transects as compared to six-transect houses. Birds under experimental pen conditions were shown to travel longer distances in larger enclosures (18, 20) with longer total and net distances, and longer mean and maximum step length (19). Eight-transect houses were not larger in total available area, but they were wider as compared to six-transect houses. The higher number of transects in wider houses and the additional time required to assess eight-transect houses resulted in birds traveling longer distances, which explains our results, especially considering the tendency of birds to move laterally when perceiving an approaching human. Our results also showed that the movement patterns are affected by the position of the transect in which the marked bird was observed (wall or central), as birds first detected at walls crossed more than two transects in both six- and eight-transect houses (Table 3). On the contrary, the mean number of transects traveled by birds located in central transects was 1.5. Birds at walls can only escape toward the central house area; therefore, the ability of moving only in one direction would explain the difference in results.

Our findings on detection and repetition rates and number of traveled transects were demonstrated on a healthy marked subpopulation, whose ability to move along transects should be better than that of unhealthy birds. When conducting welfare assessments, the interest is focused on birds with impaired welfare (e.g. lame, immobile, sick individuals). Differences in activity levels between impaired and healthy birds were demonstrated (32), not only for chickens with leg difficulties (33–35) but also for those infected with diseases (36). Welfare assessment of impaired birds with the transect method is likely to result in much lower repetition rates as birds with compromised welfare are expected to move less (if at all) and, therefore, will be less likely to be found again in the following conducted transects. Future studies using the transect method should focus on estimating these variables on an impaired subpopulation, although it is challenging as such birds should be culled by the farmer on the basis of minimizing animal suffering.

### Marked Subpopulation Distribution

The results on the distribution index suggest that the marked subpopulation was closer to a random distribution in six-transect than in eight-transect houses (see Table 4). When comparing central and wall transects, the distribution index showed opposite patterns in six- and eight-transect houses. While it tended to zero at walls in six-transect houses, it was slightly higher in central transects. The distribution index was much higher at walls for eight-transect houses. In fact, the number of observed birds doubled the expected value on walls, altering significantly the distribution index in eight-transect houses. These results may be due to two different factors. On the one hand, the wall effect (37, 38) may have been much stronger in wider eight-transect houses. Ventura et al. (39) showed a lower use of central areas in experimental small control pens when compared with pens equipped with barrier perches. Therefore, it is suggested that in larger houses, the strong preference for walls may have resulted in higher values of distribution index. On the other hand, the layout of eight-transect houses was such that the wall transects were smaller than the average with a mean width of 1.136 m. This might explain the low number of expected birds (estimated according to the transect width) in comparison to the observed number. In addition to the narrower width of wall transects, eight-transect houses were shorter than six-transect houses, resulting in a lower wall space available per unit of area (the mean percentages of wall area were 9% and 6.6% in six and eight-transect houses, respectively). Therefore, the lower availability of wall areas might have resulted in a higher demand of wall space due to stronger preference (37, 38). Such lower wall availability, combined with the above-mentioned preference for walls, may have resulted in birds congregating at walls in eight-transect houses. Although this effect was only observed in eight-transect houses, the potential for wall effects suggests that in order to have a more representative sample of the flock, the transect method should be conducted by selecting a wall and a central transect.

Flock density did not affect any of the tested variables. Although thinning resulted in densities reaching as low as 11 birds/m^2^ in some flocks, no differences were shown neither on the detection and repetition rates, nor on the number of traveled transects and distribution index. Even though Ventura et al. (39) demonstrated higher activity levels at low densities (8 and 13 birds/m^2^), they also reported a significant decrease in activity levels with age. As birds in our study were assessed at 30 days of age, when activity levels is significantly lower (37, 38, 40), this might explain the lack of effect of flock density on the number of traveled transects. Indeed, the high percentage of repetition between adjacent transects might confirm our assumption, suggesting that due to lower activity levels, birds escaping the observer did not move far away. Our findings suggest that welfare assessments using the transect method are not affected by lowering densities that resulted from thinning flocks.

### Bootstrapping Simulations

The bootstrapping technique is an analytical method designed to calculate the minimum sample effort required without losing accuracy of the sampling (41). Our results indicate that the mean percentage of detected birds remained stable even when assessing a single transect, as compared to the assessment of the entire house. Variability around the sample mean also remained stable but was slightly lower when assessing two transects in comparison to assessing only one (Table 6). These findings are in agreement with the results from Marchewka et al. (7), who reported a stable mean estimation and minor changes in SE when evaluating only 20% of the house area, equivalent in their study to two transects. Despite the fact that assessments in our study were conducted on healthy marked birds, whose ability to escape from the observer is expected to be higher than that of birds with welfare issues, SE did not increase excessively when the number of sampled transects decreased. Therefore, the results of this study are in agreement with those of the previous studies by Marchewka et al. (7, 8), who suggested assessing two transects for a representative assessment of the population. In addition, consideration should be given to the economic cost and time constraints when assessing broiler welfare in commercial flocks. Reducing sampling to two transects (wall and central) would still provide a reliable mean, minimize bird disturbance, and reduce cost and time requirements as compared to sampling the entire house. It also has the benefit of minimizing the risk of repeating birds if conducted transects are separated by at least three transects in between.

Overall, this study aimed at analyzing the robustness of the transect method using capture–recapture techniques of a marked bird subpopulation and tracking their movements under commercial conditions. Our findings were generally as expected and might be considered robust. We found higher repetition rates in six-transect houses and during morning samplings. More transversal movement was registered in wider eight-transect houses and when birds were first detected at walls. These findings are consistent with previous results, confirming that population movement under commercial conditions might potentially influence assessment outcomes. Therefore, it is recommended to skip three transects after evaluating the first one to minimize risks of repetition (if enough transects are available in the house). We found significant differences in the distribution index between central and wall areas especially in wider eight-transect houses, which was related to the higher preference for wall regions of the house. Therefore, it is advisable to sample both wall and central transects for a representative welfare assessment of the impaired population. Bootstrapping transect data showed that assessing two transects would provide comparable results to those obtained when assessing the entire house. Lower repetition rates, required time for assessment and bird disturbance, would be achieved with such recommendations while maintaining the robustness of final results.

## Data Availability

The data sets generated for this study can be found here: https://figshare.com/s/ae6b28a6bbc2b25b816d.

## Ethics Statement

This study complied with the Spanish legislation regarding the use of animals for experimental and other scientific purposes (Real Decreto 1201/2013).

## Author Contributions

IE, XA, and NB conceptualized the study. Data collection was done by NB. Formal analysis, investigation, and methodology were done by NB, XA, and IE. Supervision was handled by IE and XA. Writing including original draft preparation was handled by NB. Writing including reviewing and editing was handled by NB, IE, and XA.

### Conflict of Interest Statement

The authors declare that the research was conducted in the absence of any commercial or financial relationships that could be construed as a potential conflict of interest.
